# Metabolic and Structural Imaging at 7 Tesla After Repetitive Mild Traumatic Brain Injury in Immature Rats

**DOI:** 10.1177/1759091418770543

**Published:** 2018-05-09

**Authors:** Emin Fidan, Lesley M. Foley, Lee Ann New, Henry Alexander, Patrick M. Kochanek, T. Kevin Hitchens, Hülya Bayır

**Affiliations:** 1 Safar Center for Resuscitation Research, Department of Critical Care Medicine, University of Pittsburgh, PA, USA; 2 Pittsburgh NMR Center for Biomedical Research, Carnegie Mellon University, PA, USA; 3 Animal Imaging Center, University of Pittsburgh, PA, USA; 4 Children's Neuroscience Institute

**Keywords:** repetitive, mild head injury, neuroimaging, children, magnetic resonance imaging, diffuse tensor imaging

## Abstract

Mild traumatic brain injury (mTBI) in children is a common and serious public health problem. Traditional neuroimaging findings in children who sustain mTBI are often normal, putting them at risk for repeated mTBI (rmTBI). There is a need for more sensitive imaging techniques capable of detecting subtle neurophysiological alterations after injury. We examined neurochemical and white matter changes using diffusion tensor imaging of the whole brain and proton magnetic resonance spectroscopy of the hippocampi at 7 Tesla in 18-day-old male rats at 7 days after mTBI and rmTBI. Traumatic axonal injury was assessed by beta-amyloid precursor protein accumulation using immunohistochemistry. A significant decrease in fractional anisotropy and increase in axial and radial diffusivity were observed in several brain regions, especially in white matter regions, after a single mTBI versus sham and more prominently after rmTBI. In addition, we observed accumulation of beta-amyloid precursor protein in the external capsule after mTBI and rmTBI. mTBI and rmTBI reduced the N-acetylaspartate/creatine ratio (NAA/Cr) and increased the myoinositol/creatine ratio (Ins/Cr) versus sham. rmTBI exacerbated the reduction in NAA/Cr versus mTBI. The choline/creatine (Cho/Cr) and (lipid/Macro Molecule 1)/creatine (Lip/Cr) ratios were also decreased after rmTBI versus sham. Diffusion tensor imaging findings along with the decrease in Cho and Lip after rmTBI may reflect damage to axonal membrane. NAA and Ins are altered at 7 days after mTBI and rmTBI likely reflecting neuro-axonal damage and glial response, respectively. These findings may be relevant to understanding the extent of disability following mTBI and rmTBI in the immature brain and may identify possible therapeutic targets.

## Introduction

Recent Centers for Disease Control and Prevention (CDC) reports estimate that approximately 1.6 to 3.8 million traumatic brain injuries (TBIs) occur in the United States each year with as many as half of them unreported. Of these, 70% to 90% are classified as mild TBI (mTBI; Faul et al., 2010; Prins and Giza, 2012; CDC, 2015) commonly caused by sports injuries, accidents, and falls. Over half of the reported TBI cases occur in individuals under 19 years of age and involve critical periods in brain development (CDC, 2015). Reports show that 16% of children will sustain a concussion or mTBI by the age of 10 years (Langlois et al., 2006), and 14% of children suffering mTBI have persistent symptoms 3 months after the injury (Barlow et al., 2010). This high incidence is also reflected by hospital admission rates (Faul et al., 2010). Germane to our investigation, there is a lack of sensitive diagnostic methods for mTBI that puts children at risk of having repetitive mTBI (rmTBI). The risk of rmTBI is highest within the first 7 to 10 days after injury with 80% to 92% of repeat injuries occurring during this time period (Guskiewicz et al., 2003; McCrea et al., 2009). In addition to acute effects such as impaired memory, confusion, and headache from rmTBI, reports from autopsies of professional athletes have shown them to be suffering from chronic traumatic encephalopathy with symptoms including neurodegeneration and tau deposition (McKee et al., 2009). Although evidence is lacking in the pediatric population regarding chronic effects of rmTBI in clinical studies, in a preclinical study, we showed that rmTBI sustained at postnatal day (PND) 18 leads to axonal injury and microglial activation in immature rat brain and is associated with long-term cognitive deficits (Fidan et al., 2016).

While there is not an exact definition of mTBI, it has been suggested that clinical signs and symptoms of concussion and mTBI overlap substantially (Prins and Giza, 2012). The Concussion in Sport Group consensus statement defined concussion as follows: (a) resultant from an impulsive force transmitted to the head that can be characterized as spontaneously resolving neurologic dysfunction with a rapid onset, which may or may not involve loss of consciousness, (b) a functional disturbance rather than a structural injury, which cannot be detected by conventional neuroimaging tools such as computed tomography and magnetic resonance imaging (MRI), and (c) resolution of symptoms that typically, but not always, follows a sequential course (McCrory et al., 2017). Acute symptoms of concussion usually resolve within 7 to 10 days (Guskiewicz et al., 2003; McCrea et al., 2009). However, a notable percentage of patients (especially children) experience prolonged symptoms (Guskiewicz et al., 2003; McCrea et al., 2009; Barlow et al., 2010). As emphasized in the Concussion in Sport Group definitions, despite symptoms, structural abnormalities are hard to detect using routine neuroimaging techniques (Kuppermann et al., 2009). In a large study, Kupperman et al. reported that less than 1% of computed tomography-imaged children who suffered a head trauma showed significant structural changes. Thus, there is a need to develop more sensitive and sophisticated neuroimaging tools to detect subtle structural and/or biochemical changes after mTBI, which might be important in the diagnosis and protection against repeat injuries (Kuppermann et al., 2009; McCrory et al., 2017).

Recently, neurometabolic and microstructural changes were demonstrated in acute and subacute periods post-mTBI using more advanced imaging techniques, such as diffusion tensor imaging (DTI), and magnetic resonance spectroscopy (MRS) (Lin et al., 2012; Shenton et al., 2012). DTI differs from conventional imaging in that it maps the directional diffusion of water molecules and is sensitive to microstructural changes, such as traumatic axonal injury (Shenton et al., 2012). Quantitative measures of DTI include fractional anisotropy (FA) as well as axial diffusivity (AD), radial diffusivity (RD) and mean diffusivity (MD). FA is a scalar measure between 0 and 1, which is an indication, of the degree of diffusional anisotropy and is sensitive to microstructural changes. A value of 0 indicates isotropic diffusion in all directions, and a value of 1 is found when diffusion is restricted to only one axis. MD is a measure of total diffusion within a voxel and it is sensitive to cellularity, edema and tissue necrosis. AD and RD are directional diffusion coefficients parallel and perpendicular to the principle axis. AD will increase in white matter tracts with brain maturation and is sensitive to axonal injury, RD is sensitive to axonal diameter and demyelination (Kubicki et al., 2007). In the pediatric population, experimental studies are missing. Furthermore, reported DTI data in clinical studies are conflicting. Several clinical studies performed in children reported increases in FA and decreases in diffusivity (Wilde et al., 2008; Chu et al., 2010; Wu et al., 2010; Mayer et al., 2012; Wilde et al., 2012) pointing toward the possibility of cellular edema acutely after mTBI. On the contrary, others showed no change or a decrease in FA and an increase in diffusivity, suggesting loss of white matter due to mTBI (Roberts et al., 2014). Supporting this, Shenton and others reported decreased FA and increased MD as most commonly reported DTI abnormalities in clinical studies involving adults with mTBI (Shenton et al., 2012; Khong et al., 2016). Experimental studies in adult mTBI and rmTBI animal models also support these observations in human subjects (Li et al., 2011; Bennett et al., 2012; Donovan et al., 2014; Kamnaksh et al., 2014).

MRS is another modality that is used to detect subtle changes in neurochemical metabolites after injury (Lin et al., 2012; Gardner et al., 2014). Major metabolites examined in human and animal TBI studies include N-acetylaspartate (NAA), creatinine (Cr), choline (Cho), and myoinositol (Ins). NAA is one of the most abundant molecules in the brain (Duarte et al., 2012), and it is mostly detected in neurons (Mountford et al., 2010), thus decreases in NAA levels are considered to indicate loss or damage of neurons or neuronal processes (Smith et al., 1998; Mountford et al., 2010). NAA is synthesized in neuronal mitochondria from aspartic acid and acetyl-coenzyme A. Decreases in NAA levels correlate with the degree of neuronal mitochondrial dysfunction, which can be observed after pediatric TBI (McKenna et al., 2015). Creatinine is used as the internal standard to measure other metabolites and is assumed to be stable (Gardner et al., 2014). Choline-containing metabolites are essential for the biosynthesis of acetylcholine and for membrane lipids (Duarte et al., 2012). Myoinositol is another metabolite which is measured and considered as a marker of gliosis or inflammation (Duarte et al., 2012). MRS has been utilized in adult clinical and experimental mTBI studies (Smith et al., 1998; Vagnozzi et al., 2008; Aaen et al., 2010; Vagnozzi et al., 2010; Li et al., 2011; Maugans et al., 2012; Gardner et al., 2014; Sivak et al., 2014; Narayana et al., 2015) showing significant decreases in NAA/Cr and NAA/Cho ratios particularly in white matter early after injury (Johnson, Gay, et al., 2012; Johnson, Zhang, et al., 2012). Compared with adult brain, the developing brain has higher brain water content and ongoing myelination, as well as an immature excitatory neurotransmitter system, posing challenges for DTI and MRS studies. Although several pediatric animal mTBI models have been developed (Prins et al., 2010; Mychasiuk et al., 2014), surprisingly, there has not been a study using MRS to investigate the neurometabolic changes in the immature brain after mTBI or rmTBI.

Using a closed skull mTBI model, we previously showed that even a single episode of mTBI resulted in axonal injury (assessed by silver staining) without overt neuronal death in the corpus callosum and external capsule accompanied by microglial activation at 7 days after the impact when the injury occurred at PND 18. A second mTBI further increased the level of axonal injury and microglial activation at 7 days after the last impact (Fidan et al., 2016). Furthermore, three mTBIs suffered 24 hr apart starting at the age of PND 18 resulted in cognitive dysfunction with long-term associative learning deficits in adulthood (Fidan et al., 2016). Our current study was undertaken to investigate whether the subtle histological changes observed in this pediatric rmTBI model can be detected by noninvasive tools such as DTI and MRS at 7 days after injury and can correlate with beta-amyloid precursor protein (β-APP) staining, a commonly used method to detect axonal damage in TBI (Johnson et al., 2013). As an essential membrane protein β-APP is normally transported along the length of the axon. It is thought that mechanical deformation of white matter tracts during trauma results in disruption of axonal transport and accumulation of β-APP in damaged axons (Johnson et al., 2013). We observed significant decreases in FA and significant increases in AD and RD in several regions of brain, especially in white matter, at 7 days after mTBI and rmTBI when rats were injured at PND 18. APP staining, however, was observed only in the external capsule. MRS of the hippocampus also showed bilateral neurometabolic changes suggestive of neuro-axonal damage.

## Material and Methods

PND 10, male Sprague Dawley rat pups, along with their lactating mothers, were purchased from Harlan and housed for 7 days before experimentation. Standard rat chow and water were provided ad libitum. Day and night cycles were set at 12 hr each, and ambient room temperature was maintained at 20°C to 22°C.

Experiments were performed exploring whether DTI and MRS can detect early microstructural changes of the brain and metabolite changes in the hippocampus, respectively, after mTBI and rmTBI. At PND 18, the rats were divided into three groups and subjected to (a) three sham insults (*n*=8), (b) one mTBI, two sham insults (*n*=9, mTBI), or (c) three mTBIs (*n*=10, rmTBI) 24 hr apart. This study was approved by the University of Pittsburgh Animal Care and Use Committee.

### Mild Traumatic Brain Injury

**Figure 1.**
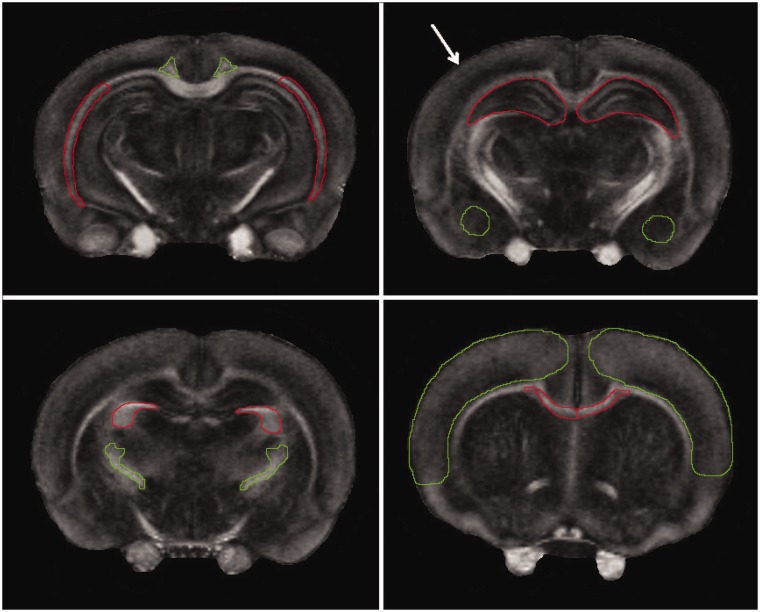
Representative axial FA images of ex vivo sham animal displaying the regions where the ROI’s were taken from. Upper left: (−4.80 mm from bregma) external capsule (red), cingulum (green). Upper right: (−3.80 mm from bregma) hippocampus (red), amygdala (green). Lower left: (−2.56 mm from bregma) fimbria (red), internal capsule (green). Lower right: (+0.20 mm from bregma) corpus callosum (red), cortex (green). The impact site for CCI is shown by white arrow.

An established model of immature mTBI (Fidan et al., 2016) was used for all experiments. PND 18 pups were anesthetized with 2% isoflurane in 2:1 N_2_O/O_2_ administered via a nose cone. Following anesthesia, rats were secured into a stereotaxic frame using ear bars, their heads were shaved, and betadine was applied. After midline scalp incision, an impact to a predefined injury site (1.8 mm caudal to bregma and 3.0 mm left of the midline, Figure 1) on the intact skull was delivered via a pneumatically driven piston. Specifically, a 9.5 mm diameter rubber ball, mounted to a bowl-shaped metal tip manufactured by the University of Pittsburgh Machine Shop, was lowered to a depth of 1.0 mm and an angle of 23°. The impact velocity was 4.0 ± 0.2 m/s over a 50 ms duration. Skin was sutured following impact, and rats were recovered in pure oxygen by nose cone with a warming pad before being returned to their cages. Sham animals underwent the same procedures, including anesthesia and surgery, with the exception of impact.

### *In Vivo* Magnetic Resonance Imaging

*In vivo* MRI studies were performed on a 7-Tesla Bruker Biospec AVIII spectrometer (Billerica, MA, USA) 7 days after the last sham insult or mTBI. Animals were placed onto a cradle with a stereotaxic head holder. Anesthesia was induced with 2% isoflurane via (1:1) N_2_O/O_2_ gas through a nose cone. Body temperature was maintained at 37.0 ± 0.5°C with warm air, controlled and monitored via a rectal temperature probe (SA Instruments Inc., Stony Brook, NY, USA).

Following pilot images, single voxel ^1^H spectroscopy was carried out using water signal suppression with variable power radiofrequency pulses with optimized relaxation delays (VAPOR; Tkac et al., 1999). Outer volume suppression combined with point-resolved spectroscopy (PRESS; Price and Arata, 1996) sequence from a 2 × 2 × 2 mm^3^ voxel placed in the hippocampal area was used for signal acquisition, with TE/TR = 40/1800 msec, spectral bandwidth = 3 kHz, number of data points = 2,048, number of averages = 576. Localized shimming was performed individually for both the ipsilateral and contralateral sides with a field map calculation that optimized first- and second-order shim values, yielding a water line width of 10 to 12 Hz. Only the hippocampus was assessed to minimize the overall examination time.

After *in vivo* spectroscopy, shimming was performed over the entire brain, and an *in vivo* DTI data set was collected using a multi-slice Echo Planar Imaging-DTI sequence with 5 reference A0 images and 30 noncollinear diffusion-weighted images with the following parameters: TE/TR = 22/7000 msec, 2 averages, 4 segments, matrix size = 128 × 128, field of view = 25.6 × 25.6 mm, 11 slices, slice thickness = 1 mm, *b* value = 1000 s/mm^2^, and Δ/δ = 10/5 msec. If significant motion artifacts were found in the scans, the anesthesia was adjusted, and scans were repeated. Total examination time for each animal was less than 110 min.

### *Ex Vivo* MRI

Following *in vivo* MRI acquisition, rats were anaesthetized using 3% isofluorane in a 2:1 N_2_O/O_2_ and transcardially perfused, first with 5% heparinized saline and then with 2% paraformaldehyde and then sacrificed by decapitation. Brains were harvested and post fixed in 2% paraformaldehyde for 24 hr following the time of sacrifice. An *ex vivo* DTI data set covering the entire brain was collected using a multi-slice spin echo sequence with the following parameters: TE/TR = 22/2500 msec, 2 averages, matrix size = 192 × 192, field of view = 19.2 ×19.2 mm, 22 slices, slice thickness = 1 mm, *b* value =1200 s/mm^2^, and Δ/δ = 12/6 ms with 22 diffusion directions and two reference A_0_ images.

### Data Analysis

The 1D proton spectra were analyzed off-line using MestReNova 10.0 software (Mestrelab Research SL, Spain). The spectral peaks from NAA, Cho, Cr, Ins, and lipid (Lip) were fitted to Gaussian lines shapes and integrated. The peak at 0.9 ppm (lip/MM1) was considered to be a combined peak of lipid and macromolecules (MM), specifically MM1, even though a longer TE was used during acquisition, we could not definitively attribute the peak to be solely lipid (Povazan et al., 2015). Results are expressed as the following metabolite ratios: NAA/Cr, Cho/Cr, NAA/Cho, Ins/Cr, and (Lip/MM1)/Cr. DSI Studio (http://dsi-studio.labsolver.org/) was used to analyze DTI data. Diffusion imaging data were fitted to yield FA, AD, RD, and MD. 

### Assessment of Histological Outcome

Following *ex vivo* MRI, brains were sectioned for histology. For cryoprotection, brains were submersed in 15% and then 30% sucrose solutions. Brains were frozen in liquid nitrogen and stored at −80°C until 40 µm sections were cut using a Leica Jung CM1800 cryotome. Free floating sections were stored in cryoprotectant at −20°C.

#### Immunohistochemistry

For APP immunohistochemistry staining, antigen retrieval was performed on four transverse 40 µm free floating sections, ∼200 µm apart spanning from ∼ −3.0 mm to −4.5 mm relative to bregma, with Antigen Decloaker solution (Biocare Medical, Concord, CA, USA) before sections were placed in 2% H_2_O_2_ for 20 min and blocked with 3% normal goat serum in Tris-buffered saline (TBS) containing 0.25% Triton X (TBS-X) for 1 hr. Sections were incubated overnight in TBS-X solution containing rabbit polyclonal beta-amyloid antibody (1:2000, Thermo-Fisher Scientific) and 1.5% normal goat serum; 24 hr later, sections were rinsed with TBS and incubated for 1 hr with anti-rabbit HRP-conjugated secondary antibody (ABC Elite kit, PK6100, Vector Laboratories, Burlingame, CA). Following incubation, sections were rinsed via TBS and stained with diaminobenzidine for visualization (diaminobenzidine, Vector Laboratories, Burlingame, CA).

### Statistical Analysis

Statistical analyses for DTI and MRS data were performed using GraphPad Prism software (GraphPad Software, Inc., La Jolla, CA). One way analysis of variance with Tukey’s post hoc analysis was used for data analyses. Statistical significance was set at *p* < .05, and all data are expressed as mean ± standard deviation.

## Results

**Figure 2.**
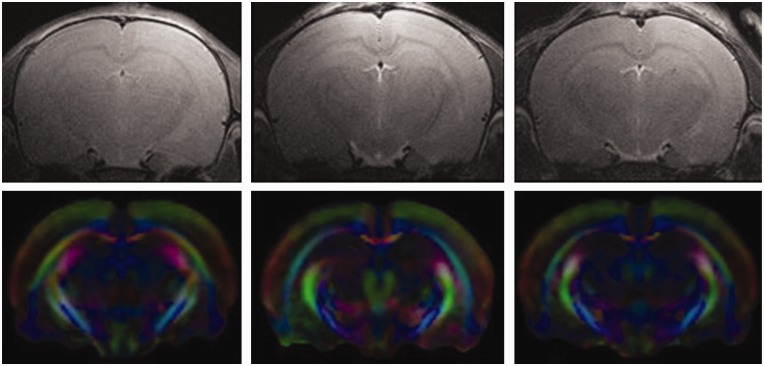
Representative *in vivo* images. Top row, T_2_-weighted anatomical images for sham (left), mTBI (middle), and rmTBI (right). Bottom row are directionally encoded color maps for sham (left), mTBI (middle), and rmTBI (right). Colors represent fiber direction, red is left/right, green is dorsal/ventral, and blue is rostral/caudal.

We observed significant changes in both DTI and MRS indices after a single or repetitive mTBI. Regions of interests for each hemisphere, including the corpus callosum, cortex, cingulum, external capsule, and hippocampus, were analyzed from all slices containing the regions of interest using assignments from a rat brain atlas (Paxinos and Watson, 1986). Representative regions of interest are shown using *ex vivo* FA images in Figure 1. Representative *in vivo* T_2_ and directionally encoded color (DEC) maps of sham, mTBI, and rmTBI rat brains are shown in Figure 2. Tables 1 to 4 list the differences seen between groups with *in vivo* DTI, specifically changes in FA, RD, AD, and MD in the regions of interests 7 days post injury. *Ex vivo* FA measures are shown in Table 5. While a single insult resulted in changes in FA and MD compared with sham, significant changes in all four DTI parameters were observed after rmTBI.

**Table 1.** In vivo Fractional Anisotropy in Rat Brain After Sham Injury, Single or Repeated mTBI.

				*p*		
Region of interest	Sham	mTBI	rmTBI	Sham vs. mTBI	Sham vs. rmTBI	mTBI vs. rmTBI
I Corpus callosum	0.48±0.01	0.45±0.02	0.40±0.01	**.036**	**<.001**	**<.001**
C Corpus callosum	0.47±0.01	0.45±0.04	0.42±0.04	.23	**.027**	.29
I Cingulum	0.40±0.01	0.39±0.01	0.34±0.02	.56	**<.001**	**<.001**
C Cingulum	0.38±0.01	0.41±0.01	0.39±0.04	.12	.50	.31
I Fimbria	0.46±0.02	0.48±0.03	0.49±0.02	.48	.09	.63
C Fimbria	0.48±0.04	0.49±0.02	0.49±0.03	.47	.58	.86
I Internal capsule	0.51±0.03	0.53±0.03	0.52±0.03	.47	.55	.78
C Internal capsule	0.48±0.02	0.50±0.02	0.48±0.04	.20	.97	.34
I Hippocampus	0.19±0.02	0.13±0.02	0.12±0.01	**<.001**	**<.001**	.79
C Hippocampus	0.17±0.04	0.14±0.04	0.15±0.03	.52	.23	.63
I Cortex	0.27±0.02	0.25±0.02	0.24±0.01	.15	**.005**	.37
C Cortex	0.26±0.03	0.25±0.02	0.25±0.02	.53	.24	.64
I External capsule	0.42±0.01	0.40±0.01	0.36±0.01	**.034**	**<.001**	**<.001**
C External capsule	0.39±0.02	0.41±0.02	0.39±0.02	.03	.53	.15
I Amygdala	0.19±0.02	0.20±0.02	0.20±0.03	.70	.72	.96
C Amygdala	0.18±0.03	0.22±0.04	0.18±0.02	.69	.66	.091

*Note.* I = ipsilateral or impact side; C = contralateral or uninjured side; mTBI = mild traumatic brain injury; rmTBI = repeated mild traumatic brain injury. *N* for groups are Sham = 6, mTBI = 5, and rmTBI = 8. Results presented as *M* ± *SD*. Significant differences are shown in bold.

*p* < .05 accepted as statistically significant and shown in bold.

**Table 2.** In Vivo Radial Diffusivity in Rat Brain After Sham Injury, Single or Repeated mTBI.

				*p*		
Region of interest	Sham	mTBI	rmTBI	Sham vs. mTBI	Sham vs. rmTBI	mTBI vs. rmTBI
I Corpus callosum	0.64±0.01	0.66±0.04	0.70±0.01	.25	**<.001**	**.03**
C Corpus callosum	0.68±0.04	0.65±0.04	0.68±0.04	.25	.74	.37
I Cingulum	0.65±0.02	0.63±0.02	0.65±0.02	.08	.70	.20
C Cingulum	0.65±0.03	0.62±0.02	0.62±0.03	.06	.18	.92
I Fimbria	0.68±0.02	0.67±0.03	0.65±0.03	.40	**.04**	.29
C Fimbria	0.67±0.02	0.64±0.02	0.64±0.015	.07	**.027**	.97
I Internal capsule	0.54±0.02	0.53±0.03	0.53±0.02	.56	.26	.82
C Internal capsule	0.56±0.02	0.54±0.02	0.55±0.02	.13	.15	.36
I Hippocampus	0.75±0.01	0.77±0.01	0.79±0.02	.21	**<.001**	**.017**
C Hippocampus	0.78±0.03	0.76±0.01	0.77±0.03	.26	.37	.77
I Cortex	0.69±0.01	0.68±0.01	0.68±0.01	.35	.36	.91
C Cortex	0.69±0.01	0.68±0.01	0.68±0.01	.55	.40	.82
I External capsule	0.64±0.01	0.64±0.01	0.67±0.02	.97	**.005**	**.01**
C External capsule	0.65±0.01	0.65±0.01	0.68±0.01	.61	**.001**	**<.001**
I Amygdala	0.82±0.05	0.78±0.04	0.79±0.05	.12	.20	.70
C Amygdala	0.79±0.04	0.78±0.02	0.79±0.02	.37	.99	.17

*Note.* I = ipsilateral or impact side; C = contralateral or uninjured side; mTBI = mild traumatic brain injury; rmTBI = repeated mild traumatic brain injury. Results presented as *M* ± *SD*. *N* for groups are Sham = 6, mTBI = 5, and rmTBI = 8. Significant differences are shown in bold.

*p* < .05 accepted as statistically significant and shown in bold.

**Table 3.** In Vivo Axial Diffusivity in Rat Brain After Sham Injury, Single or Repeated mTBI.

				*p*		
Region of interest	Sham	mTBI	rmTBI	Sham vs. mTBI	Sham vs. rmTBI	mTBI vs. rmTBI
I Corpus callosum	1.30±0.01	1.34±0.07	1.39±0.02	.24	**<.001**	.12
C Corpus callosum	1.36±0.10	1.34±0.08	1.30±0.08	.70	.74	.37
I Cingulum	1.18±0.03	1.18±0.06	1.15±0.07	.97	.41	.47
C Cingulum	1.16±0.04	1.17±0.04	1.14±0.06	.58	.58	.37
I Fimbria	1.51±0.05	1.49±0.12	1.51±0.09	.81	.88	.91
C Fimbria	1.53±0.11	1.52±0.06	1.51±0.09	.74	.54	.76
I Internal capsule	1.32±0.04	1.35±0.03	1.32±0.07	.20	.95	.43
C Internal capsule	1.28±0.04	1.30±0.02	1.26±0.07	.44	.46	.26
I Hippocampus	0.93±0.01	0.94±0.04	0.98±0.02	.80	**.007**	**.037**
C Hippocampus	0.98±0.08	0.94±0.05	0.94±0.05	.29	.16	.81
I Cortex	0.98±0.01	1.00±0.03	1.06±0.02	.40	**<.001**	**<.001**
C Cortex	1.03±0.06	1.00±0.02	1.01±0.03	.26	.42	.39
I External capsule	1.21±0.01	1.22±0.02	1.28±0.02	.44	**<.001**	**<.001**
C External capsule	1.21±0.01	1.21±0.03	1.18±0.05	.61	.15	.21
I Amygdala	1.09±0.03	1.05±0.02	1.06±0.04	.10	.17	.65
C Amygdala	1.02±0.05	1.07±0.02	1.05±0.06	.13	.45	.36

*Note.* I = ipsilateral or impact side; C = contralateral or uninjured side; mTBI = mild traumatic brain injury; rmTBI = repeated mild traumatic brain injury. Results presented as *M* ± *SD*. *N* for groups are Sham=6, mTBI=5, and rmTBI=8. Significant differences are shown in bold.

*p*<.05 accepted as statistically significant and shown in bold.

**Table 4.** In Vivo Mean Diffusivity in Rat Brain After Sham Injury, Single or Repeated mTBI.


				*p*		
Region of interest	Sham	mTBI	rmTBI	Sham vs. mTBI	Sham vs. rmTBI	mTBI vs. rmTBI
I Corpus callosum	0.90±0.02	0.86±0.02	0.86±0.03	**.015**	**.02**	.82
C Corpus callosum	0.91±0.03	0.88±0.01	0.89±0.04	.14	.23	.93
I Cingulum	0.83±0.02	0.81±0.03	0.82±0.03	.24	.39	.71
C Cingulum	0.82±0.03	0.80±0.02	0.80±0.03	.26	.13	.70
I Fimbria	0.96±0.02	0.95±0.06	0.94±0.04	.84	.10	.61
C Fimbria	0.96±0.03	0.93±0.03	0.93±0.03	.25	.53	.73
I Internal capsule	0.80±0.02	0.80±0.02	0.80±0.03	.84	.53	.48
C Internal capsule	0.80±0.02	0.79±0.01	0.79±0.02	.29	.11	.50
I Hippocampus	0.83±0.03	0.82±0.01	0.82±0.03	.67	.63	.91
C Hippocampus	0.85±0.03	0.82±0.01	0.82±0.03	.11	.15	.93
I Cortex	0.80±0.02	0.78±0.01	0.79±0.02	.30	.34	.78
C Cortex	0.80±0.03	0.79±0.01	0.79±0.01	.27	.33	.61
I External capsule	0.85±0.02	0.84±0.01	0.84±0.03	.15	.20	.88
C External capsule	0.86±0.03	0.84±0.01	0.84±0.03	.10	.13	.98
I Amygdala	0.91±0.04	0.87±0.03	0.88±0.05	.09	.24	.58
C Amygdala	0.87±0.03	0.88±0.01	0.88±0.03	.72	.68	.90

*Note.* I = ipsilateral or impact side; C = contralateral or uninjured side; mTBI = mild traumatic brain injury; rmTBI = repeated mild traumatic brain injury. Results presented as *M* ± *SD*. *N* for groups are Sham=6, mTBI=5, and rmTBI=8. Significant differences are shown in bold.

*p*˂.05 accepted as statistically significant and shown in bold.

**Table 5.** *Ex Vivo* FA Results in Rat Brain After Sham Injury, Single or Repeated mTBI.

				*p*		
Region of interest	Sham	mTBI	rmTBI	Sham vs. mTBI	Sham vs. rmTBI	mTBI vs. rmTBI
I Corpus callosum	0.51±0.03	0.44±0.03	0.40±0.01	**<.001**	**<.001**	**.033**
C Corpus callosum	0.52±0.04	0.50±0.06	0.40±0.01	.52	**<.001**	**.001**
I Cingulum	0.40±0.03	0.38±0.01	0.34±0.01	.54	**.004**	**.040**
C Cingulum	0.39±0.03	0.41±0.01	0.40±0.02	.32	.74	.37
I Fimbria	0.48±0.03	0.48±0.03	0.49±0.02	.85	.50	.67
C Fimbria	0.48±0.01	0.49±0.03	0.49±0.02	.72	.21	.78
I Internal capsule	0.49±0.04	0.48±0.02	0.46±0.03	.86	.30	.26
C Internal capsule	0.48±0.03	0.49±0.01	0.49±0.02	.76	.54	.65
I Hippocampus	0.19±0.05	0.14±0.01	0.12±0.01	**.017**	**.001**	.462
C Hippocampus	0.19±0.06	0.18±0.01	0.16±0.01	.55	.17	.060
I Cortex	0.27±0.02	0.26±0.02	0.23±0.02	.67	**.012**	.067
C Cortex	0.27±0.01	0.27±0.02	0.27±0.03	.58	.77	.91
I External capsule	0.41±0.02	0.37±0.02	0.33±0.02	**.006**	**<.001**	**.001**
C External capsule	0.42±0.02	0.43±0.04	0.42±0.02	.71	.71	.86
I Amygdala	0.20±0.02	0.20±0.02	0.21±0.02	.72	.69	.47
C Amygdala	0.21±0.02	0.20±0.01	0.20±0.01	.28	.30	.84

*Note.* I = ipsilateral or impact side; C = contralateral or uninjured side; mTBI = mild traumatic brain injury; rmTBI = repeated mild traumatic brain injury. Results presented as *M* ± *SD*. *N* for groups are Sham=6, mTBI=6, and rmTBI=7. Significant differences are shown in bold.

*p*<.05 accepted as statistically significant and shown in bold.

Table 1 shows the changes in *in vivo* FA in sham, mTBI and rmTBI. FA decreased in the ipsilateral corpus callosum, hippocampus, and external capsule following mTBI versus sham, and this decrease was more pronounced after rmTBI. The ipsilateral cingulum and cortex also saw decreased FA after rmTBI compared to sham. Surprisingly, the contralateral corpus callosum also showed a significant decrease in FA in rmTBI animals when compared to sham.

Table 5 shows changes in *ex vivo* FA of sham, mTBI and rmTBI groups. Similar to the *in vivo* DTI results, *ex vivo* FA levels decreased in the ipsilateral corpus callosum, hippocampus, and external capsule after single mTBI versus sham, and these decreases were more prominent in the rmTBI group. Ipsilateral cingulum and cortex were additional areas of decreased FA after rmTBI versus sham. The contralateral corpus callosum had significantly decreased FA in the rmTBI group.

**Figure 3.**
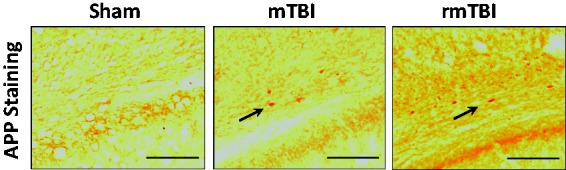
APP staining 7 days after repeated sham insult, mTBI, or rmTBI in external capsule. Representative pictures (scale bar = 100 µm) of coronal sections at approximately –3.0 to 4.5 mm, bregma. APP staining is indicated by arrows. mTBI = mild traumatic brain injury; rmTBI = repeated mild traumatic brain injury; APP = amyloid precursor protein.

While a single episode of mTBI did not result in changes in RD (Table 2) or AD (Table 3) in any region of interest, rmTBI, on the other hand, resulted in increases in RD and AD levels in the ipsilateral corpus callosum versus sham; and in the ipsilateral hippocampus and ipsilateral external capsule compared to sham and mTBI animals. Furthermore, the contralateral external capsule showed a significant increase in RD while in the ipsilateral and contralateral fimbria we observed a decrease in RD following rmTBI 7 days after the last impact, when compared with sham (Table 2). The only significant change in MD was observed in the ipsilateral corpus callosum in both the mTBI and rmTBI groups versus sham (Table 4). Histological analysis of the brain showed a sparse accumulation of APP only in the ipsilateral external capsule (Figure 3).

**Figure 4.**
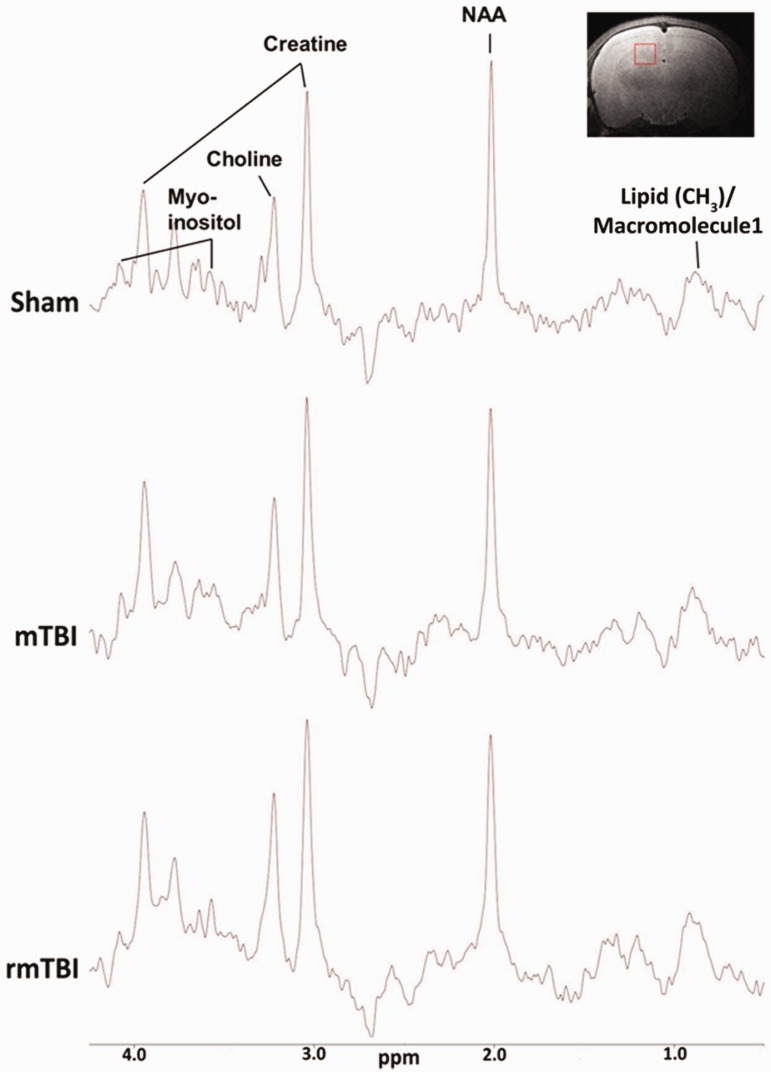
Proton magnetic resonance spectra representation acquired at magnetic field strength of 7 Tesla. The main individual metabolite peaks are labeled, with the *x* axis representing chemical shift in parts per million (ppm) and *y* axis indicating amplitude or concentration of the chemical. mTBI = mild traumatic brain injury; rmTBI = repeated mild traumatic brain injury; NAA = N-acetylaspartate.

**Figure 5.**
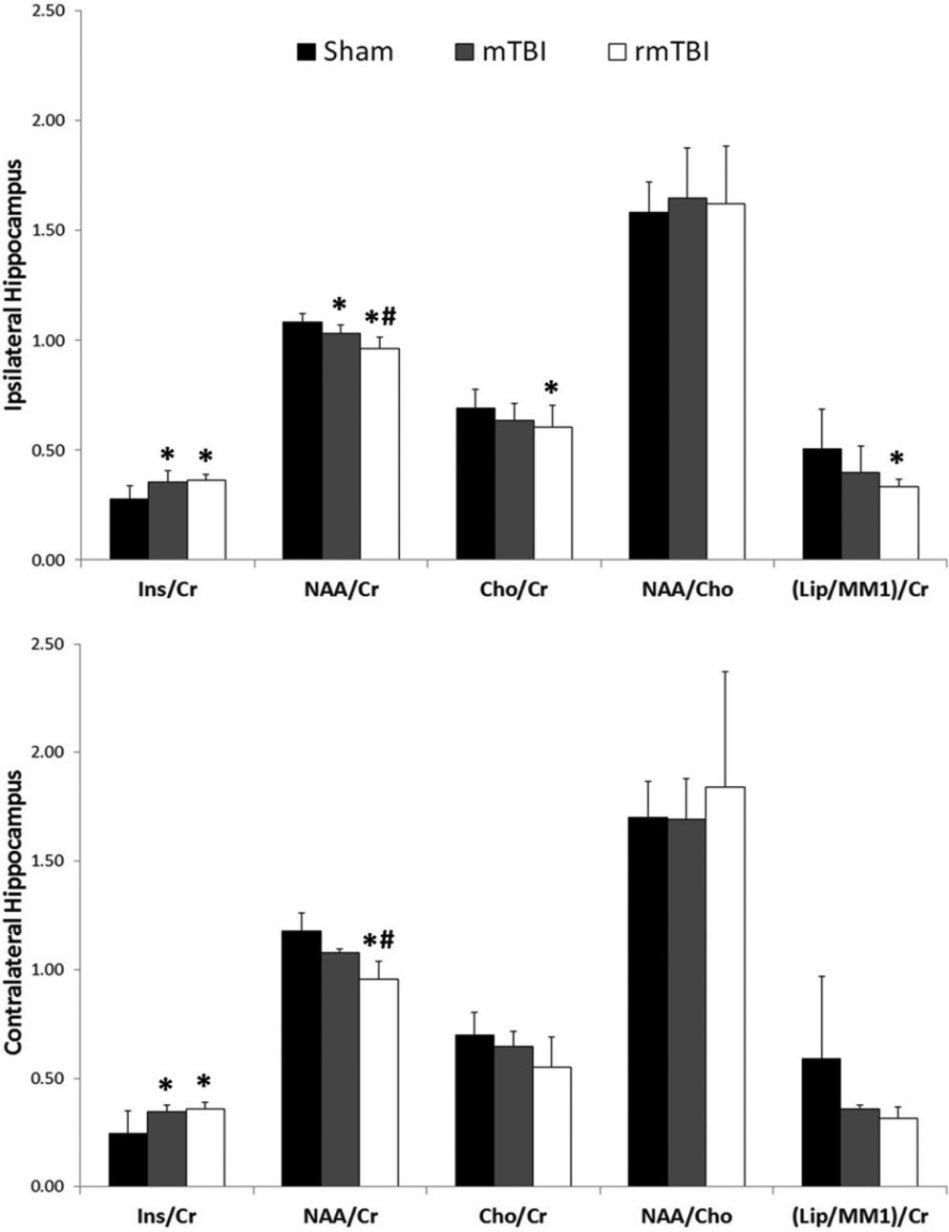
Metabolite concentration ratios in ipsilateral (top panel) and contralateral (bottom panel) hippocampus 7 days after the last sham or mTBI. NAA = N-acetylaspartate; Ins = myoinositol; Cr = creatinine; Cho = choline; (Lip/MM1) = lipid/macromolecule1); mTBI = mild traumatic brain injury; rmTBI = repeated mild traumatic brain injury. *p < .05 versus Sham, ^#^*p* < .05 versus mTBI.

Representative MR spectra from sham, mTBI, and rmTBI groups are shown in Figure 4, as well as an example of where the voxel was positioned within the hippocampus. Analysis of this data obtained from ipsilateral and contralateral hippocampi (Figure 4, insert) of rats 7 days after the last impact revealed significant differences between groups. NAA/Cr ratio in the ipsilateral hippocampus was significantly reduced after mTBI and rmTBI, and the Ins/Cr ratio was significantly increased in both injury groups compared with sham. There was a small, but significant, further reduction in the NAA/Cr after rmTBI versus mTBI. Cho/Cr and Lip/Cr ratios in the ipsilateral hippocampus were also significantly decreased after rmTBI. NAA/Cho was not significantly different between injured and sham rats (Figure 5, top panel). Measurements from the contralateral hippocampus revealed a significant reduction in NAA/Cr ratio levels of the rmTBI group versus sham and mTBI. In addition, both mTBI and rmTBI groups showed a significant increase in Ins/Cr ratios compared with sham controls (Figure 5, bottom panel).

## Discussion

Mild TBI in children is a common and serious public health problem. Traditional neuroimaging findings are often normal in children who sustain mTBI putting them at risk for repeat injury. More sensitive imaging techniques capable of detecting subtle alterations in neurophysiology after injury are needed. We examined neurochemical and white matter changes in immature rat brain after mTBI and rmTBI using DTI, proton MRS, and APP immunohistochemistry. Our findings indicate axonal membrane damage and glial proliferation primarily in the external capsule, corpus callosum, and hippocampus, after mTBI and to a greater extent after rmTBI. Our data correlate with traumatic axonal injury, microglial activation, and neurobehavioral deficits we previously observed in this model (Fidan et al., 2016). To our knowledge, this is the first study combining DTI and MRS imaging *in vivo* in the immature brain after mTBI or rmTBI. Our results suggest that combining DTI and MRS might improve the success of detecting microstructural changes *in vivo* after mTBI (Shenton et al., 2012; Narayana et al., 2015).

### Decreased Anisotropy and Increased Diffusivity After mTBI and rmTBI in the Immature Brain

Diffuse axonal damage is considered as a signature pathology of TBI regardless of severity of the injury (Buki and Povlishock, 2006). Traumatic axonal injury is characterized by widespread damage primarily to axons in the brainstem, corpus callosum, parasagittal white matter of cerebrum, and the gray-white matter junctions of the cerebral cortex (Meythaler et al., 2001). Although, the exact reason for this vulnerability is not known, it is likely due to biomechanical effects of the primary injury and resultant biochemical and cellular secondary injury cascades (Kochanek et al., 2000). Mechanisms of traumatic axonal injury are complex and have been widely studied (Buki and Povlishock, 2006). Following primary injury, interruption of axonal transport leads to accumulation of organelles and proteins such as APP (Figure 2) resulting in axonal retraction ball formation and disruption (Buki and Povlishock, 2006; Fidan et al., 2016). This process peaks at 24-hr post injury but might continue for weeks to months (Prins and Giza, 2012; Fidan et al., 2016) resulting in Wallerian degeneration (Buki and Povlishock, 2006). Axonal injury might decrease local diffusion anisotropy and lamination due to demyelination thus resulting in decreases in FA and increases in diffusivity measures such as RD and AD in subacute stages of injury (Song et al., 2002; Li et al., 2011). Using the same model utilized in the current study, we previously showed (Fidan et al., 2016) that rats exposed to single mTBI and rmTBI displayed prominent axonal silver staining indicating traumatic axonal injury in the external capsule underlying the impact site and in the corpus callosum—the same areas where we observed decreases in FA and increases in RD and AD using *in vivo* and *ex vivo* DTI in the current study. The extent of injury is primarily in the ipsilateral hemisphere, indicating the effect of localized impact. Significant decreases in the FA were observed mostly in the ipsilateral hemisphere with the exception of contralateral corpus callosum. The reasons for this could be due to the nature of the impact. Previous studies utilizing a unilateral mouse closed head injury mTBI model found similar findings with contralateral corpus callosum being the only area with FA changes on DTI imaging (Bennett et al., 2012). It is likely that the functional and structural organization of corpus callosum connecting the two hemispheres predisposes expansion of unilateral changes to contralateral region. Given the reports that silver staining might be underrepresenting the extent of axonal pathology after TBI (Johnson et al., 2013), we used APP staining to further explore axonal injury in this follow-up study. Accumulation of APP was observed only in the ipsilateral external capsule (Figure 2). It is possible that the time point we chose, 7 days after injury, is relatively late to detect the most robust APP accumulation. It was shown that APP accumulation can be identified within hours after injury in the damaged axons (Johnson et al., 2013). Bennett et al. (2012) also reported detectable DTI changes without significant APP accumulation at 24 hr and 7 days after rmTBI.

While this is the first *in vivo* preclinical DTI study in the immature brain after mTBI, significant decreases in FA measurements in the corpus callosum, brain stem, hippocampus, and several other white matter regions were reported at 24-hr post injury in an adult mTBI model (Li et al., 2011). These changes in FA levels were persistent in almost all regions of interest at 7 days post injury. Axial diffusivity however was found to have decreased within the first 5 days after the injury. By 7 days, AD normalized and RD was increased compared with uninjured controls. The authors suggest that axonal injury could increase the restriction on diffusion parallel to the main axis of the ﬁbers and decrease local diffusion anisotropy (Li et al., 2011). Mac Donald et al. (2007) also reported similar decreases in AD levels during the acute stages that normalized 1 month after the injury. Our findings differ from these two studies likely due to differences in age of the animals, injury severity, and time point of imaging. A more severe injury in the other models might have resulted in increased more abundant neuroinflammation and significant decreases in AD levels in the acute stages post injury of post injury which might resolve at a later time point (Yu et al., 2017). Wallerian degeneration secondary to axonal injury along with resolving edema might also explain the later increases observed in AD (Thuen et al., 2009). We previously showed that mTBI resulted in traumatic axonal injury without overt neuronal death in the corpus callosum and external capsule accompanied by inducible nitric oxide synthase (iNOS) positive proinflammatory microglial activation at 7 days after the impact (Fidan et al., 2016). Future studies utilizing DTI longitudinally at acute and subacute time points in the immature brain might be more revealing in understanding the temporal changes in FA, AD, and RD.

We and others have shown that repeat insults after a mTBI worsen traumatic axonal injury and neurobehavioral deficits in adult and pediatric models (Prins et al., 2010; Donovan et al., 2014; Fidan et al., 2016; Mannix et al., 2017; Yu et al., 2017). Consistent with this, our DTI data showed that rmTBI exacerbated the changes in FA and other diffusivity indices when compared to a single episode of mTBI. Several studies in adult rmTBI models support our findings. Donovan et al. (2014) showed that rmTBI resulted in significant increases in AD and RD in the corpus callosum compared with sham and mTBI 6 months after injury by using *ex vivo* DTI. In another study, Yu et al. (2017) showed that rmTBI lead to decreases in FA and AD in the corpus callosum 42 days after injury, correlating with microglial activation in the cortical region under the impact.

Decreased FA and increased diffusivity have been the most commonly reported DTI abnormalities in adults after clinical or experimental mTBI (Li et al., 2011; Shenton et al., 2012; Donovan et al., 2014; Khong et al., 2016). DTI studies in pediatric mTBI have been performed, up until now', only in adolescent human subjects and the characterization of anisotropic abnormalities during the subacute period have been controversial (Wilde et al., 2008; Chu et al., 2010; Wu et al., 2010; Wilde et al., 2012; Roberts et al., 2014). A number of factors including injury severity, instrumental conditions, patient characteristics, time of imaging after injury, and analysis methods can affect the results of DTI and MRS. In addition, imaging in the developing brain poses additional challenges compared with adult brain due to high brain water content, ongoing myelination, and the lack of maturity of the excitatory neurotransmitter system along with a higher tendency to have cerebral edema (Kochanek, 2006; Ferrazzano et al., 2013). Contrary to our findings, several studies in the pediatric population show increases in FA and decreases in diffusivity (Wilde et al., 2008; Chu et al., 2010; Mayer et al., 2012) while others report no significant differences in DTI parameters after mTBI (Maugans et al., 2012). Mayer et al. reported increased FA and decreased RD in children, even ∼4 months after injury. The authors suggested that the most plausible explanation for these findings could be cytotoxic edema (Mayer et al., 2012). While this might be a reasonable explanation for acute stages of injury, since it is known that the immature brain has a higher tendency to develop cytotoxic and vasogenic edema (Adelson and Kochanek, 1998), most imaging studies were performed between 3 days and 1 week after injury, and in some cases even 4 months after a concussion (Mayer et al., 2012). The age of patients, type and location of injury, recall bias, small sample size, and developing myelination of the brain are some of the factors that might explain the heterogeneity of these results.

### Changes in the Neurometabolites After mTBI and rmTBI in the Immature Brain

MRS has recently been used to detect neurometabolic changes after a concussion. The metabolites mostly reported are NAA, Cr, Cho, Ins, and lactate. In this study, MRS experiments were focused on the hippocampus since it plays an important role in learning and memory and it is known to be selectively vulnerable to TBI (Otani et al., 2011). It is likely that regions other than hippocampi experience neurometabolic changes after mTBI and rmTBI. Future studies utilizing MRS imaging could reveal important information on metabolic changes in different regions of the brain after mTBI. Our previous study, using the same injury model, showed impaired learning and memory retention in rats after three mTBIs 24 hr apart starting at the age of PND 18 (Fidan et al., 2016). Focusing just on the hippocampal region also decreased the time that these immature animals were exposed to anesthesia. We observed that the NAA/Cr ratio in the ipsilateral hippocampus was significantly reduced after mTBI and rmTBI, and the Ins/Cr ratio was significantly increased in both injury groups compared with sham. These data suggest reduced neuronal health (NAA/Cr) and enhanced glial proliferation (Ins/Cr) after single or repeat injury. There was a small, but significant, further reduction in the NAA/Cr after rmTBI versus mTBI showing repeated impacts result in further neuronal damage. Cho/Cr and Lip/Cr ratios in the ipsilateral hippocampus were also significantly decreased after rmTBI suggesting axonal damage.

Studies in pediatric populations using MRS after mTBI or rmTBI are lacking. On the other hand, several MRS studies performed in children following moderate to severe TBI reported consistent reduction of NAA, even years after injury, associated with neurological or neurocognitive outcomes (Walz et al., 2008; Babikian et al., 2010). Interestingly and contrary to our results, elevation of Cho levels was observed early after TBI normalizing at later time points (Babikian et al., 2010). Choline has been suggested as a biomarker of ongoing pathological processes such as demyelination or an inflammatory response.

Several studies in adults who suffered from sport-related concussion showed significant decreases in the NAA/Cr and NAA/Cho ratio especially in the white matter early after the impact (Henry et al., 2011; Johnson, Gay, et al., 2012). In athletes who were asymptomatic 3 days after a single concussion, MRS showed significant decreases (%18.5 vs. control group) in the NAA/Cr ratio 3 days after injury with a modest recovery (3%) at 15 days and a full recovery 30 days post injury. Interestingly three subjects who suffered another concussion within 15 days of the previous concussion showed persistent decreases in the NAA/Cr ratio at 30 days with recovery at 45 days after first injury (Vagnozzi et al., 2008). Thus the enhanced vulnerability of the brain to repeat insults might be due to mTBI-induced neurometabolic disturbance, which can persist weeks after the injury (Giza and Hovda, 2001). Furthermore, changes in MRS were shown to correlate with postconcussion symptom resolution. Sustained alterations in the NAA/Cr and NAA/Cho ratio at 30 days after a single concussion were observed when the resolution of clinical symptoms were prolonged (between 11 and 19 days; Vagnozzi et al., 2010). By using a rotational head injury model, Smith et al. (1998) also showed decreases in NAA levels which persisted up to 7 days after injury in pigs. In line with these findings, we have observed significant decreases in the hippocampal NAA/Cr ratio after a single injury that was exacerbated with repeated insults. While a single impact did not result in significant decreases in the Cho/Cr and (lipid/MM1)/Cr ratios, rmTBI caused significant decreases in the ratios of both metabolites.

## Conclusions

We observed that DTI and ^1^H-MRS can identify subtle structural and metabolic alterations after mTBI and rmTBI in the immature brain. NAA and Ins are altered after mTBI and rmTBI likely reflecting neuro-axonal cell damage and glial response, respectively. The DTI findings and APP accumulation along with the decrease in Cho and Lip after rmTBI may reflect damage to axonal membranes. These findings may be relevant to understanding the extent of disability following mTBI and rmTBI in the immature brain. These abnormalities may also represent possible therapeutic targets. Future studies combining longitudinal advanced neuroimaging with behavioral testing could further our understanding of the pathophysiology of mTBI and assess the vulnerable period for excerbation of injury following rmTBI in the immature brain.

## Summary

Diffusion tensor imaging and magnetic resonance spectroscopy are useful in detecting subtle changes resulting from mild TBI which may be relevant to understanding the extent of disability following mTBI and rmTBI in the immature brain and may represent therapeutic targets.
